# Supplementary material for: Clostridioides difficile LuxS mediates inter-bacterial interactions within biofilms
Source: Sci Rep. 2019 Jul 9;9:9903. doi: 10.1038/s41598-019-46143-6 (PMC6616478; doi:10.1038/s41598-019-46143-6)
Supplement: Supplementary file 1 — Supplementary data [file 41598_2019_46143_MOESM1_ESM.pdf]

Clostridioides difficile LuxS mediates inter-bacterial interactions within biofilms

Ross T. Slater[1], Lucy R. Frost[1], Sian E. Jossi[1], Andrew D. Millard[2] and Meera Unnikrishnan[1]

[1]University of Warwick, Gibbet Hill Road, Coventry, United Kingdom CV4 7AL

[2]University of Leicester, University Road, Leicester LE1 7RH, UK

Running title: Role of *C. difficile* LuxS in biofilms

[*]Correspondence: Meera Unnikrishnan, Microbiology and Infection Group, Division of Biomedical Sciences, Warwick Medical School, University of Warwick, Coventry, CV4 7AL, UK, Email: m.unnikrishnan@warwick.ac.uk

KEYWORDS**:** *C. difficile*, *B. fragilis*, biofilms, luxS, quorum sensing

Supplementary Figure Legends

Figure S1. Spore counts from *C. difficile WT* and *luxS* biofilms.

Bacterial counts were determined on BHIS or BHIS + 0.1% sodium taurocholate from biofilm cultures with and without heat treatment (65°C for 25 mins) after 24 h (A) and 72 h (B). Data are representative of 2 independent experiments performed in triplicates.

Figure S2. Analysis of AI-2 production by WT and *LuxS* in planktonic and biofilm growth conditions.

Strains were grown anaerobically in BHI medium. (**A**) Planktonic aliquots were removed for OD_{600} readings (line) and cell-free supernatants were collected at each time-point (0 – 12 h) and assayed for AI-2 activity using *V. harveyi* BB170 reporter assay (bars). Data shown is the mean of 3 independent experiments in triplicates and error bars indicate SD (**B**) Cell free supernatants were collected from WT and *luxS* biofilms cultured for 24 h and WT planktonic cultures at 8 h. AI-2 activity was measured using *V. harveyi* BB170 reporter assay. Bioluminescence is shown as a percentage of wildtype *V. harveyi* BB120 bioluminescence, which was assumed to be 100 %. Data are representative of 2 independent experiments performed in triplicates.

Figure S3. Dose dependent effects of DPD.

Dosage response observed for *luxS* with the addition of exogenous 4,5-dihydroxy-2,3-pentanedione (DPD) at concentrations of 1, 10, 100, and 1000 nM after 72 hr in BHIS + 0.1 M glucose. N=3, error bars show standard deviation. ****$p<0.0001$ by one-way ANOVA, Tukey's multiple comparison test.

Figure S4. Growth curve of *B. fragilis* and *C. difficile* in BHIS-G.

B. fragilis and *C. difficile* were grown overnight and diluted to a starting OD_{600} of 0.5 in fresh BHIS + 0.1 M Glucose. *C. difficile* grew faster with a logarithmic rate of 0.380 per hour and a maximal OD_{600} of 2.216, compared to *B. fragilis* which had a logarithmic rate of 0.208 per hour and a maximal OD_{600} of 1.704. N=3, error bars show standard deviation.

Figure S5. *C. difficile inhibition in mixed biofilms*

Colony counts from monoculture *C. difficile* and co-culture biofilms with *B. fragilis* after 72 h. Data is mean of 3 independent experiments done in triplicates, error bars show standard deviation. *p<0.05 Student's t-test.

Figure S6. AI-2 production in mixed biofilms

Late log-phase *C. difficile* WT is displayed as a control. Cell-free supernatants were taken from both mono and co-culture biofilms of *C. difficile* (WT and *luxS*) and *B. fragilis.* These were assayed for AI-2 activity using *V. harveyi*. Bioluminescence is shown as a percentage of wild-type *V. harveyi* BB120 bioluminescence, which was assumed to be 100%. Data is representative of 2 independent experiments. The grey line indicates the limit of detection.

Figure S7. Spore counts from mixed biofilms

Bacterial counts were determined on BHIS + 0.1% sodium taurocholate from single (*C. difficile* WT or *luxS*) or mixed (*C. difficile* WT or *luxS-B. fragilis*) biofilm cultures

with and without heat treatment (65°C for 25 mins) after 24h. Data shown are representative of 2 independent experiments.

Figure S8. Cell free *B. fragilis* supernatants do not inhibit *C. difficile*

(A) Colony counts were performed at 24 h for WT and WT resuspended in cell-free *B. fragilis* supernatant. **(B)** WT biofilms were grown for 24 h after which the supernatant was replaced with either fresh BHIS + 0.1M glucose, cell-free *B. fragilis* biofilm supernatant or cell-free co-culture biofilm supernatant. Samples were incubated for a further 24 h before colony counts were performed. Data shown is the mean of 3 independent experiments in triplicates and error bars indicate SD, $*p < 0.05$, $**p<0.01$ as determined by one-way ANOVA, Tukey's multiple comparison test.

Figure S9. Effect of trehalose on *C. difficile* WT and *luxS* biofilms

(**A**) WT was grown in BHIS + 0.1M glucose with 1 μM, 10 μM, 100 μM and 1000 μM trehalose for 72 hrs. 2000 μM Glucose was used as a control. (**B**) Both WT and *luxS* were tested with concentrations of 1 μM, 10 μM trehalose. Since yeast extract (a supplement of BHIS) contains trehalose, these samples were grown in BHI + L-Cysteine. WT grown in BHIS-G was used as for comparison. N=3, error bars represent standard deviation. Ns- non significant, $***p<0.001$, $****p<0.0001$ by one-way ANOVA, Tukey's multiple comparison test.

Figure S1

A

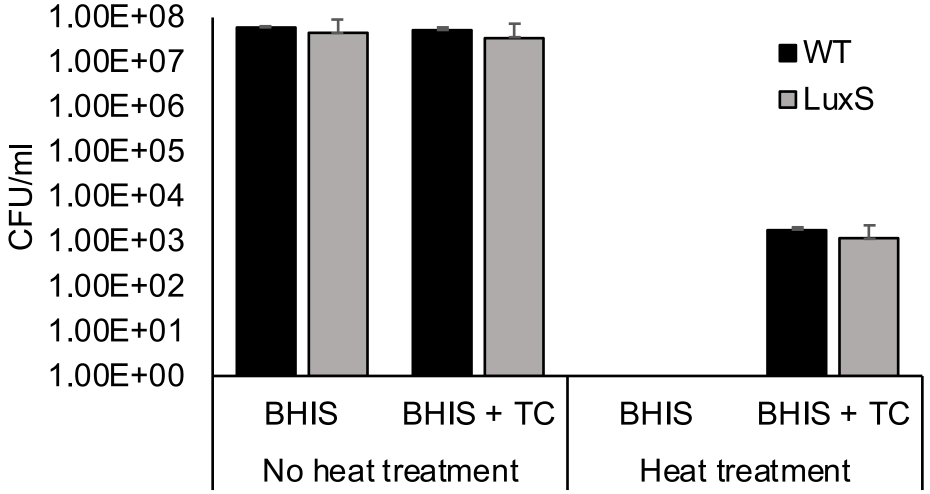

1.00E+08
1.00E+07
1.00E+06
1.00E+05
1.00E+04
1.00E+03
1.00E+02
1.00E+01
1.00E+00
CFU/ml
WT
LuxS
BHIS
BHIS + TC
BHIS
BHIS + TC
No heat treatment
Heat treatment

B

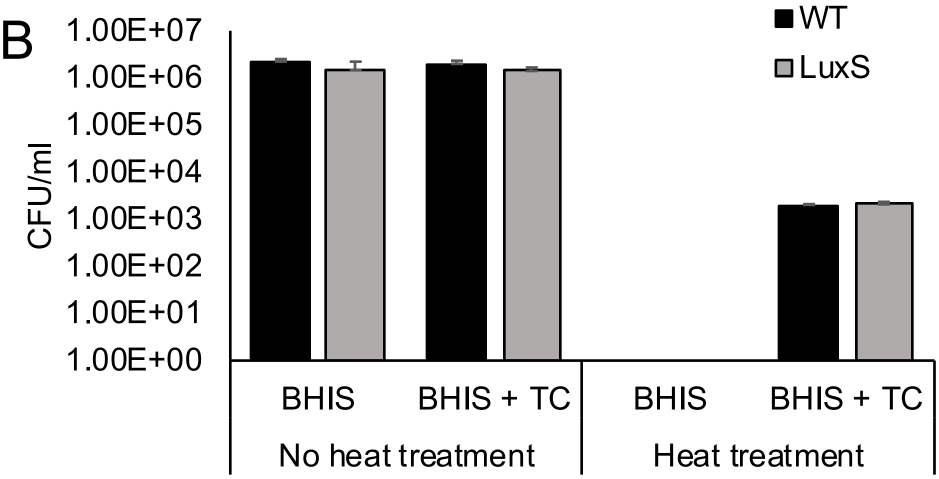

1.00E+07
1.00E+06
1.00E+05
1.00E+04
1.00E+03
1.00E+02
1.00E+01
1.00E+00
CFU/ml
WT
LuxS
BHIS
BHIS + TC
BHIS
BHIS + TC
No heat treatment
Heat treatment

Figure S2

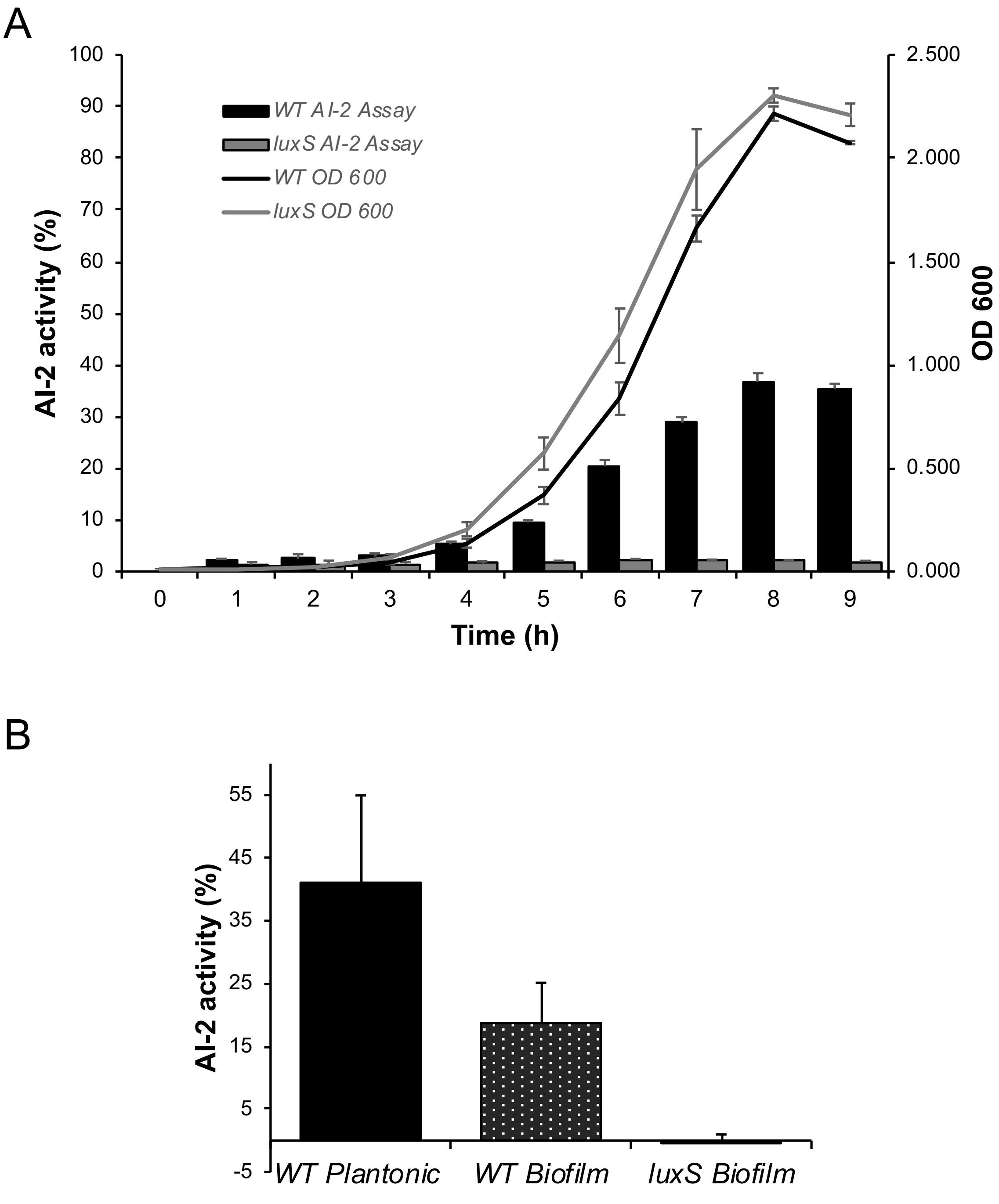
A
100
90
80
70
60
50
40
30
20
10
0
AI-2 activity (%)
2.500
2.000
1.500
1.000
0.500
0.000
OD 600
WT AI-2 Assay
luxS AI-2 Assay
WT OD 600
luxS OD 600
0 1 2 3 4 5 6 7 8 9
Time (h)
B
55
45
35
25
15
5
-5
AI-2 activity (%)
WT Plantonic
WT Biofilm
luxS Biofilm

Figure S3

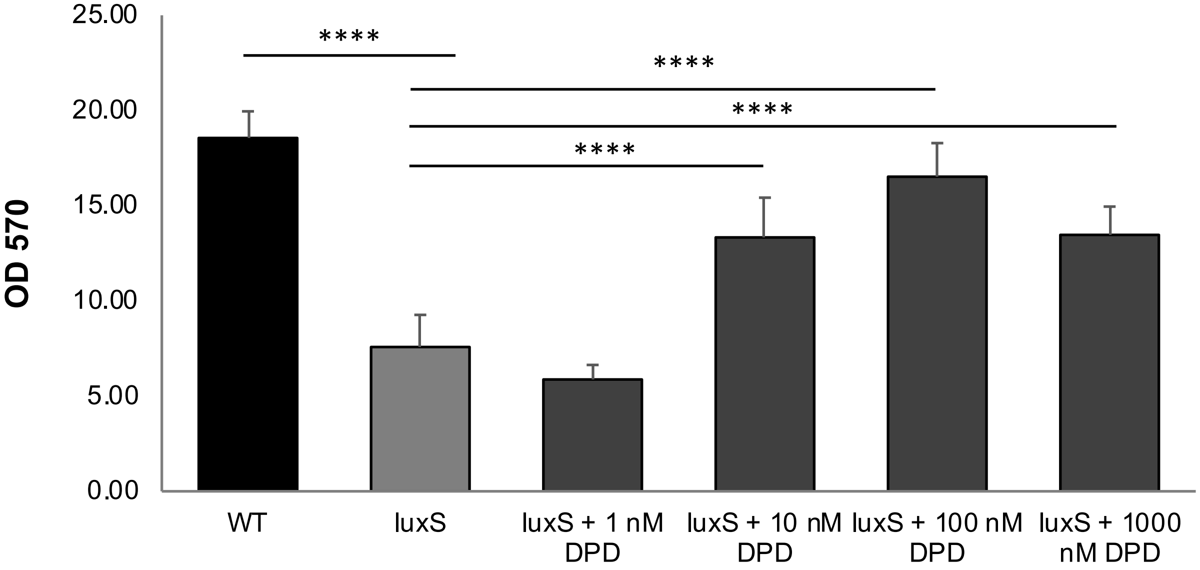
25.00
20.00
15.00
10.00
5.00
0.00
OD 570
****
****
****
****
WT
luxS
luxS + 1 nM DPD
luxS + 10 nM DPD
luxS + 100 nM DPD
luxS + 1000 nM DPD

Figure S4

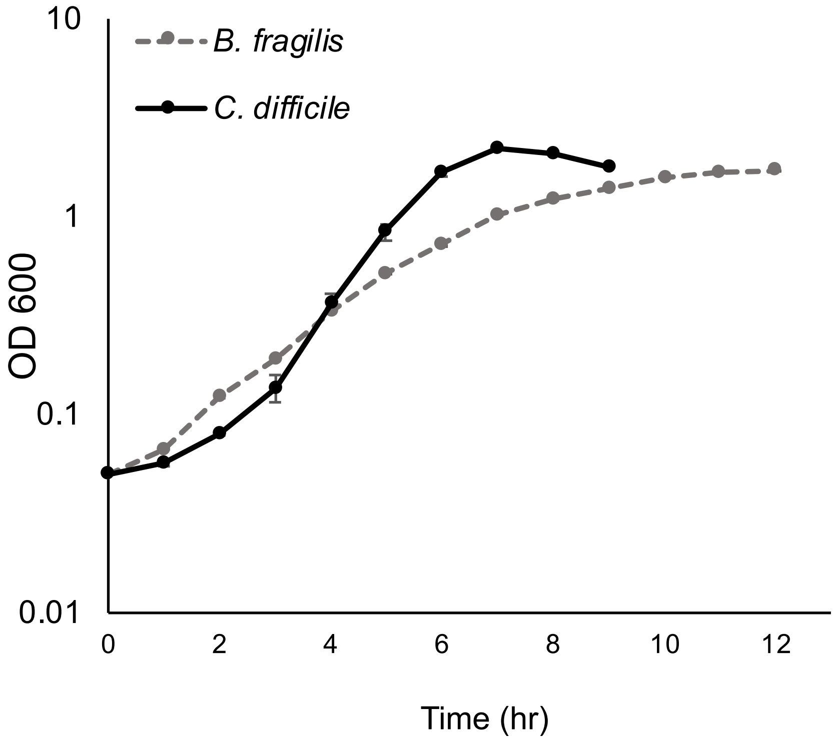

Figure S5

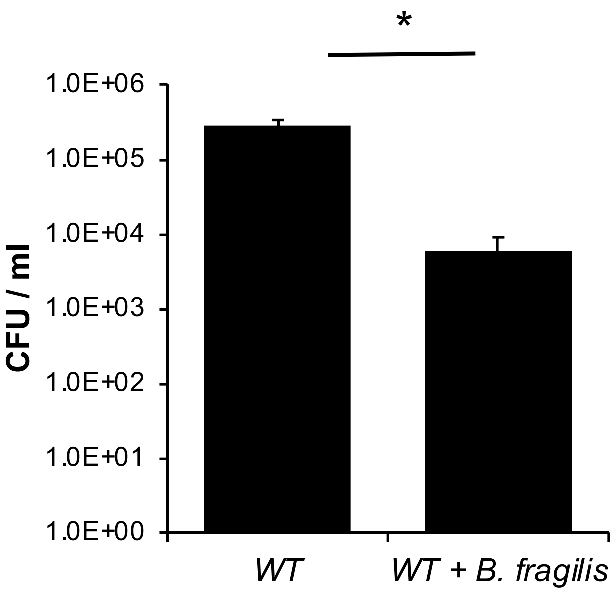
*
1.0E+06
1.0E+05
1.0E+04
1.0E+03
1.0E+02
1.0E+01
1.0E+00
CFU / ml
WT
WT + B. fragilis

Figure S6

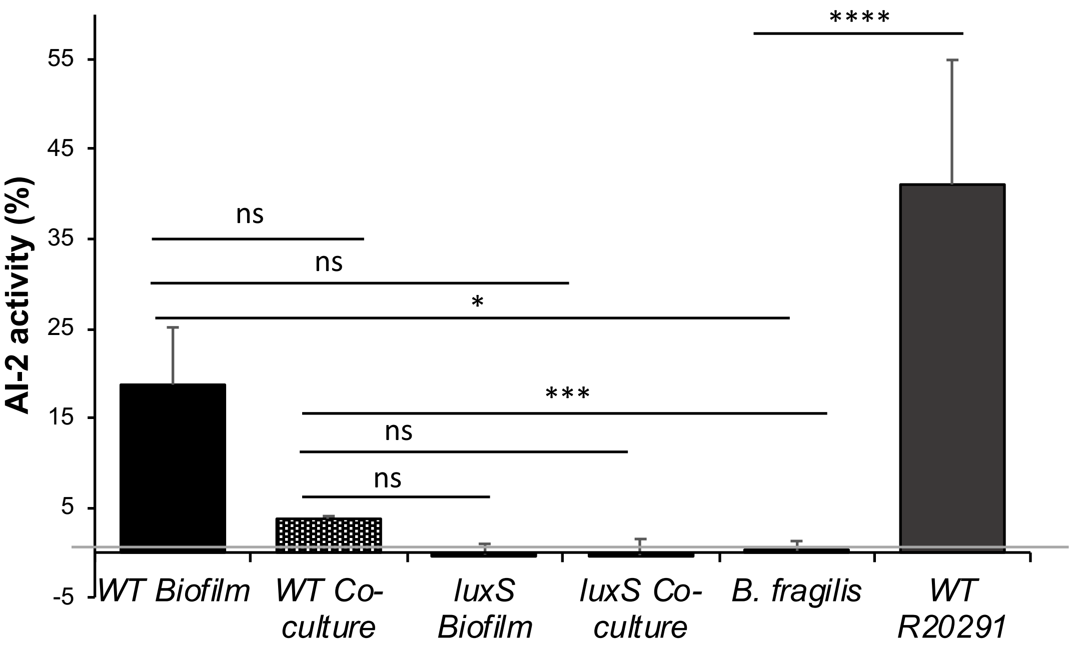
AI-2 activity (%)
55
45
35
25
15
5
-5
ns
ns
*
***
ns
ns
****
WT Biofilm
WT Co-culture
luxS Biofilm
luxS Co-culture
B. fragilis
WT R20291

Figure S7

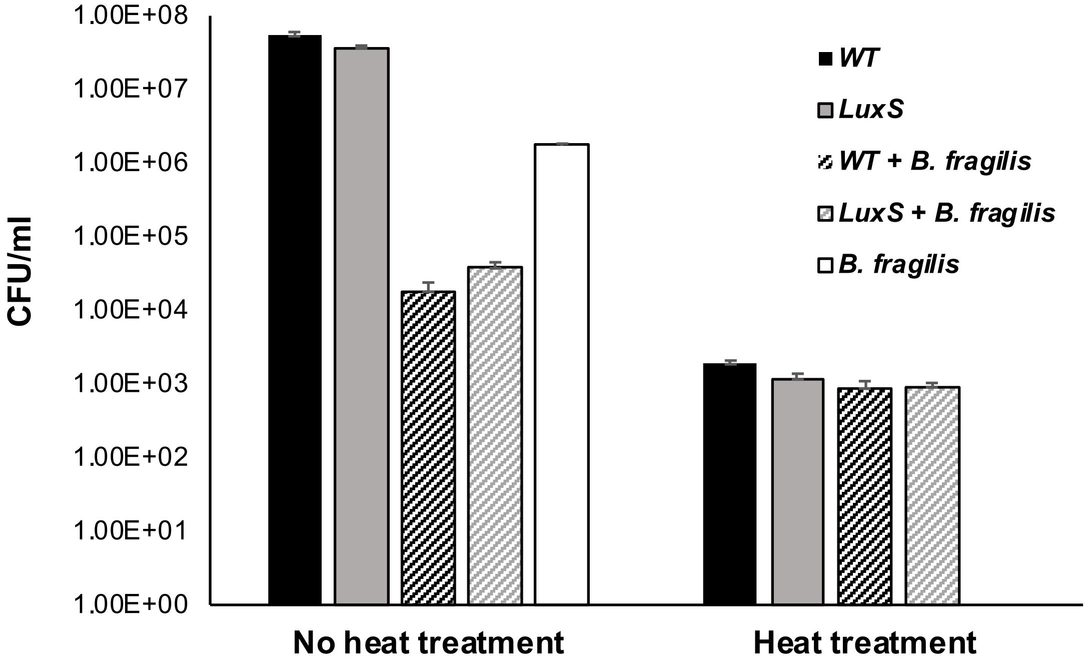
1.00E+08
1.00E+07
1.00E+06
1.00E+05
1.00E+04
1.00E+03
1.00E+02
1.00E+01
1.00E+00
CFU/ml
WT
LuxS
WT + B. fragilis
LuxS + B. fragilis
B. fragilis
No heat treatment
Heat treatment

Figure S8

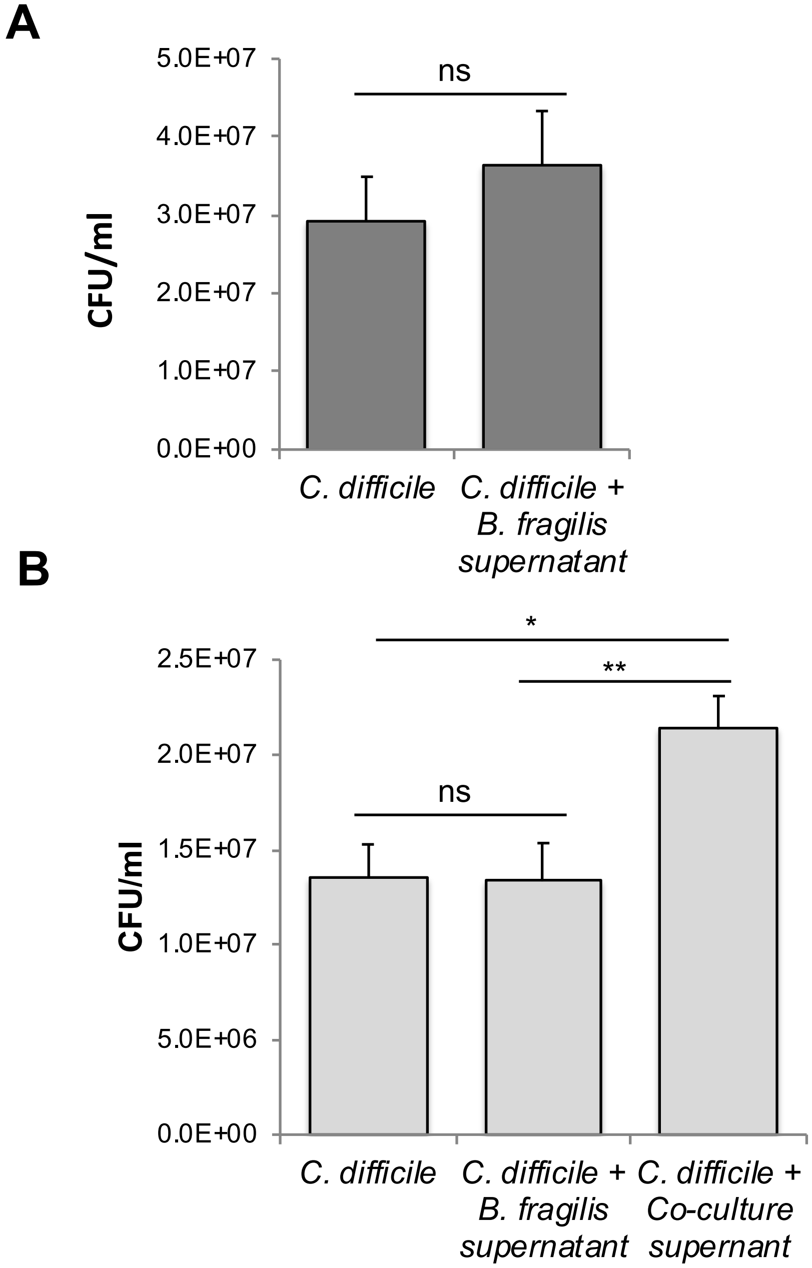
A
5.0E+07
4.0E+07
3.0E+07
2.0E+07
1.0E+07
0.0E+00
CFU/ml
ns
C. difficile
C. difficile + B. fragilis supernatant
B
2.5E+07
2.0E+07
1.5E+07
1.0E+07
5.0E+06
0.0E+00
CFU/ml
*
**
ns
C. difficile
C. difficile + B. fragilis supernatant
C. difficile + Co-culture supernant

Figure S9

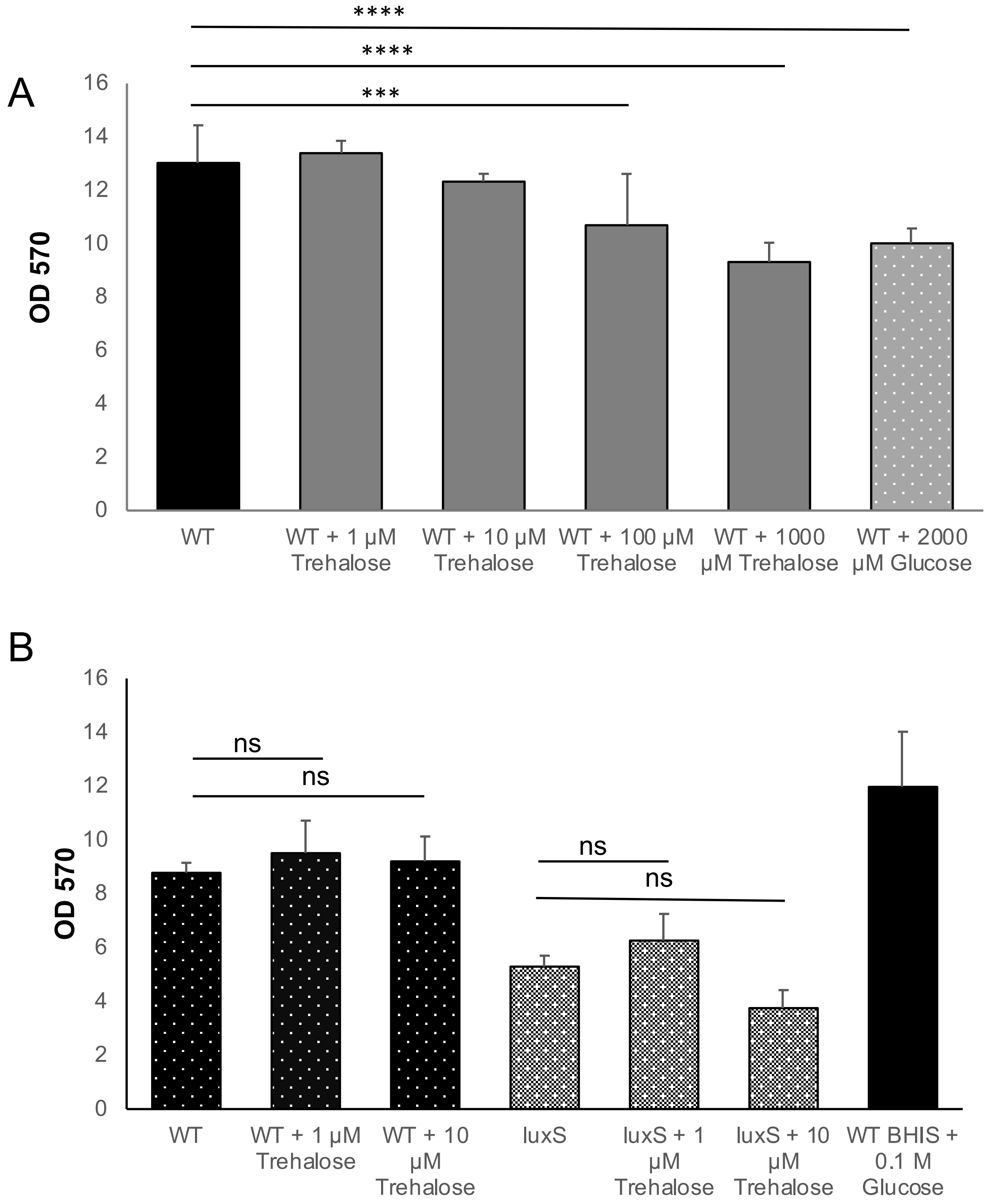
A
****
****
***
OD 570
16
14
12
10
8
6
4
2
0
WT
WT + 1 µM Trehalose
WT + 10 µM Trehalose
WT + 100 µM Trehalose
WT + 1000 µM Trehalose
WT + 2000 µM Glucose
B
ns
ns
ns
ns
OD 570
16
14
12
10
8
6
4
2
0
WT
WT + 1 µM Trehalose
WT + 10 µM Trehalose
luxS
luxS + 1 µM Trehalose
luxS + 10 µM Trehalose
WT BHIS + 0.1 M Glucose

Table S1: Genes up- and down-regulated in *B. fragilis* co-cultured with both WT and *luxS C. difficile.*

No	Gene identifier	log2FoldChange	Product
1	BF1917_00021	0.8222	hypothetical protein
2	BF1917_00043	1.67588	tRNA-Gln(ttg)
3	BF1917_00113	0.95251	Transcriptional regulatory protein CssR
4	BF1917_00148	1.47898	L-fucose isomerase
5	BF1917_00150	0.84558	Lactaldehyde reductase
6	BF1917_00221	2.16886	DNA-binding transcriptional repressor MngR
7	BF1917_00222	2.55126	hypothetical protein
8	BF1917_00365	1.42774	fec operon regulator FecR
9	BF1917_00371	1.24232	tRNA-Met(cat)
10	BF1917_00577	1.0794	HTH-type transcriptional activator Btr
11	BF1917_00591	1.24631	tRNA-Tyr(gta)
12	BF1917_00627	0.98501	Glycosyl hydrolase family 57
13	BF1917_00674	1.31953	fec operon regulator FecR
14	BF1917_00677	2.94941	hypothetical protein
15	BF1917_00753	0.99535	hypothetical protein
16	BF1917_00756	0.86105	50S ribosomal protein L25
17	BF1917_00791	1.45917	Alkyl hydroperoxide reductase subunit F
18	BF1917_00793	0.93169	Alkyl hydroperoxide reductase subunit C
19	BF1917_00811	0.95941	Outer membrane protein 41 precursor
20	BF1917_00831	0.99547	fec operon regulator FecR
21	BF1917_00843	0.85771	Alpha-xylosidase
22	BF1917_00846	1.31112	hypothetical protein
23	BF1917_00863	1.01842	Ferrous iron transport protein B
24	BF1917_00864	1.21647	hypothetical protein
25	BF1917_00865	1.03526	tRNA-Cys(gca)
26	BF1917_00893	0.82647	hypothetical protein
27	BF1917_01029	0.94956	Galactokinase
28	BF1917_01061	1.35058	indolepyruvate oxidoreductase subunit beta
29	BF1917_01086	1.16541	hypothetical protein
30	BF1917_01126	1.12128	hypothetical protein
31	BF1917_01202	1.16407	hypothetical protein
32	BF1917_01239	1.06702	Leucine Rich repeats (2 copies)
33	BF1917_01253	1.2035	UDP-glucose 4-epimerase
34	BF1917_01277	0.9888	Undecaprenol kinase
35	BF1917_01283	1.00392	ATP synthase epsilon chain
36	BF1917_01284	1.16107	hypothetical protein
37	BF1917_01305	1.61776	Sensor histidine kinase RcsC

38	BF1917_01329	0.88732	7%2C8-dihydro-6-hydroxymethylpterin-pyrophosphokinase (HPPK)
39	BF1917_01338	1.66918	tRNA-Arg(tct)
40	BF1917_01379	1.5168	tRNA-Lys(ctt)
41	BF1917_01426	1.40284	Chaperone protein HtpG
42	BF1917_01440	0.80392	LysM domain/BON superfamily protein
43	BF1917_01445	0.98821	hypothetical protein
44	BF1917_01565	1.45037	hypothetical protein
45	BF1917_01566	1.3672	transcriptional repressor DicA
46	BF1917_01568	1.24792	integration host factor subunit alpha
47	BF1917_01586	0.97287	Enamine/imine deaminase
48	BF1917_01618	2.07361	hypothetical protein
49	BF1917_01668	1.0958	ACT domain protein
50	BF1917_01699	1.01266	hypothetical protein
51	BF1917_01704	0.93246	Oxygen regulatory protein NreC
52	BF1917_01705	1.11144	hypothetical protein
53	BF1917_01783	1.19659	hypothetical protein
54	BF1917_01784	1.06401	hypothetical protein
55	BF1917_01794	1.03333	Inner membrane protein YhaI
56	BF1917_01838	0.92354	hypothetical protein
57	BF1917_01900	0.80784	Glutaconyl-CoA decarboxylase subunit beta
58	BF1917_01987	1.62881	hypothetical protein
59	BF1917_02028	1.2856	3-isopropylmalate dehydrogenase
60	BF1917_02029	1.43244	2-isopropylmalate synthase
61	BF1917_02030	1.22383	3-isopropylmalate dehydratase small subunit
62	BF1917_02031	1.22256	3-isopropylmalate dehydratase large subunit
63	BF1917_02052	1.77273	NADP-dependent 7-alpha-hydroxysteroid dehydrogenase
64	BF1917_02145	0.80664	multifunctional tRNA nucleotidyl transferase/2'3'-cyclic phosphodiesterase/2'nucleotidase/phosphatase
65	BF1917_02150	1.24574	hypothetical protein
66	BF1917_02230	0.86406	Acetolactate synthase isozyme 2 large subunit
67	BF1917_02241	1.6209	fec operon regulator FecR
68	BF1917_02252	1.04906	Methylmalonyl-CoA mutase large subunit
69	BF1917_02364	0.99688	Outer membrane protein TolC precursor
70	BF1917_02388	0.80476	Elongation factor Ts
71	BF1917_02389	1.00102	30S ribosomal protein S2
72	BF1917_02390	0.88503	30S ribosomal protein S9
73	BF1917_02399	1.54669	hypothetical protein

74	BF1917_02408	1.20627	Fimbrillin-A associated anchor proteins Mfa1 and Mfa2
75	BF1917_02411	1.43474	Carbamoyl-phosphate synthase large chain
76	BF1917_02433	1.18261	Transcriptional regulatory protein DegU
77	BF1917_02533	1.01466	30S ribosomal protein S7
78	BF1917_02534	0.85195	30S ribosomal protein S12
79	BF1917_02590	1.09676	site-specific tyrosine recombinase XerC
80	BF1917_02599	1.13134	fec operon regulator FecR
81	BF1917_02675	1.09631	Autoinducer 2 sensor kinase/phosphatase LuxQ
82	BF1917_02743	1.69888	RNA polymerase sigma factor SigV
83	BF1917_02779	1.00209	tRNA-Ser(tga)
84	BF1917_02781	0.80239	Type-1 restriction enzyme R protein
85	BF1917_02832	1.0499	23S ribosomal RNA
86	BF1917_02841	1.26941	hypothetical protein
87	BF1917_02895	1.17407	hypothetical protein
88	BF1917_02908	0.85299	putative type I restriction enzymeP M protein
89	BF1917_02926	1.35419	hypothetical protein
90	BF1917_02940	1.72848	fec operon regulator FecR
91	BF1917_02946	1.05842	Tyrosine recombinase XerC
92	BF1917_03008	1.45389	mRNA interferase YafQ
93	BF1917_03009	1.16616	hypothetical protein
94	BF1917_03031	1.47571	hypothetical protein
95	BF1917_03037	1.20593	hypothetical protein
96	BF1917_03072	5.99042	Rubrerythrin
97	BF1917_03086	7.42484	Desulfoferrodoxin
98	BF1917_03094	1.64632	hypothetical protein
99	BF1917_03132	1.26258	hypothetical protein
100	BF1917_03138	1.21443	hypothetical protein
101	BF1917_03154	1.10663	tRNA-Leu(taa)
102	BF1917_03155	9.49728	Virus attachment protein p12 family protein
103	BF1917_03166	5.22671	hypothetical protein
104	BF1917_03176	1.19636	tRNA-Cys(gca)
105	BF1917_00094	-1.0009	putative oxidoreductase UxuB
106	BF1917_00133	-1.0426	hypothetical protein
107	BF1917_00198	-1.0327	ECF RNA polymerase sigma factor SigH
108	BF1917_00199	-1.4827	hypothetical protein
109	BF1917_00200	-1.672	putative TonB-dependent receptor precursor
110	BF1917_00201	-2.2887	hypothetical protein
111	BF1917_00233	-1.5951	hypothetical protein
112	BF1917_00234	-1.7168	hypothetical protein
113	BF1917_00235	-1.6642	Periplasmic beta-glucosidase precursor

114	BF1917_00236	-1.4454	SusD family protein
115	BF1917_00237	-1.7551	TonB-dependent Receptor Plug Domain protein
116	BF1917_00238	-1.1093	hypothetical protein
117	BF1917_00253	-1.591	Cytochrome c-type protein NrfH
118	BF1917_00254	-1.4394	hypothetical protein
119	BF1917_00272	-1.2798	Glutamate decarboxylase
120	BF1917_00273	-1.0404	Glutaminase 1
121	BF1917_00311	-1.2125	Chaperone protein Skp precursor
122	BF1917_00329	-1.7304	Glutamate/gamma-aminobutyrate antiporter
123	BF1917_00330	-1.3858	hypothetical protein
124	BF1917_00331	-1.2441	hypothetical protein
125	BF1917_00332	-1.6386	Malate dehydrogenase
126	BF1917_00355	-1.4376	hypothetical protein
127	BF1917_00356	-1.3021	hypothetical protein
128	BF1917_00417	-1.0045	Outer membrane protein TolC precursor
129	BF1917_00418	-0.9189	Efflux pump membrane transporter BepE
130	BF1917_00452	-1.2843	hypothetical protein
131	BF1917_00556	-1.5284	LemA family protein
132	BF1917_00557	-1.0771	Alpha/beta hydrolase family protein
133	BF1917_00619	-1.1745	RNA polymerase sigma factor SigM
134	BF1917_00656	-1.1581	Thioredoxin reductase
135	BF1917_00665	-1.1051	ECF RNA polymerase sigma factor SigW
136	BF1917_00667	-0.8588	hypothetical protein
137	BF1917_00695	-1.033	hypothetical protein
138	BF1917_00721	-1.3608	hypothetical protein
139	BF1917_00779	-1.1636	H(+)/Cl(-) exchange transporter ClcA
140	BF1917_00796	-0.9578	hypothetical protein
141	BF1917_00797	-0.9605	hypothetical protein
142	BF1917_00804	-0.9906	5-amino-6-(5-phosphoribosylamino)uracil reductase
143	BF1917_00844	-1.1187	lipoprotein involved with copper homeostasis and adhesion
144	BF1917_00854	-1.4374	hypothetical protein
145	BF1917_00858	-1.0785	VIT family protein
146	BF1917_00878	-1.1337	Sodium-dependent dicarboxylate transporter SdcS
147	BF1917_00891	-2.0183	Porin subfamily protein
148	BF1917_00908	-1.9735	Histidine decarboxylase proenzyme precursor
149	BF1917_00930	-1.2932	Bifunctional aspartate aminotransferase and L-aspartate beta-decarboxylase
150	BF1917_00931	-1.4384	Aspartate/alanine antiporter
151	BF1917_00956	-1.1286	hypothetical protein
152	BF1917_00958	-1.3981	hypothetical protein

153	BF1917_00960	-1.4226	hypothetical protein
154	BF1917_00961	-1.862	hypothetical protein
155	BF1917_00980	-1.3254	putative inner membrane protein
156	BF1917_00989	-1.5085	DUF based on B. Theta Gene description
157	BF1917_00990	-1.542	hypothetical protein
158	BF1917_00996	-1.0481	metal-dependent hydrolase
159	BF1917_00998	-1.7058	hypothetical protein
160	BF1917_00999	-1.4252	hypothetical protein
161	BF1917_01000	-1.9487	hypothetical protein
162	BF1917_01006	-0.951	2-oxoisovalerate dehydrogenase subunit beta
163	BF1917_01090	-1.5908	ECF RNA polymerase sigma factor SigW
164	BF1917_01091	-1.9091	hypothetical protein
165	BF1917_01092	-1.6308	hypothetical protein
166	BF1917_01094	-1.1489	site-specific tyrosine recombinase XerD
167	BF1917_01143	-0.862	Glycerate dehydrogenase
168	BF1917_01153	-1.0557	LemA family protein
169	BF1917_01167	-1.2786	Collagen triple helix repeat (20 copies)
170	BF1917_01240	-1.1545	hypothetical protein
171	BF1917_01266	-1.2582	Transcriptional activator protein Anr
172	BF1917_01273	-1.0268	Dihydroorotate dehydrogenase (quinone)
173	BF1917_01274	-2.1585	Acetylxylan esterase precursor
174	BF1917_01295	-1.3227	inner membrane protein
175	BF1917_01307	-1.8784	hypothetical protein
176	BF1917_01325	-1.1429	hypothetical protein
177	BF1917_01357	-1.3565	putative peroxiredoxin
178	BF1917_01363	-1.8439	Inner membrane protein YhiM
179	BF1917_01395	-1.288	hypothetical protein
180	BF1917_01396	-1.2754	hypothetical protein
181	BF1917_01397	-0.9999	hypothetical protein
182	BF1917_01412	-1.7894	Outer membrane protein OprM precursor
183	BF1917_01413	-2.3962	Efflux pump periplasmic linker BepF
184	BF1917_01414	-1.7576	Efflux pump membrane transporter BepE
185	BF1917_01465	-0.8542	Outer membrane efflux protein
186	BF1917_01477	-1.3277	Sirohydrochlorin cobaltochelatase
187	BF1917_01486	-0.9303	Multidrug export protein EmrB
188	BF1917_01487	-1.1674	putative multidrug resistance protein EmrK
189	BF1917_01503	-1.2942	Ribonucleoside-diphosphate reductase subunit beta
190	BF1917_01543	-1.4322	hypothetical protein
191	BF1917_01544	-1.5553	hypothetical protein
192	BF1917_01562	-1.1674	acid-resistance membrane protein
193	BF1917_01610	-0.8239	Superoxide dismutase [Mn/Fe]
194	BF1917_01629	-0.8023	hypothetical protein

195	BF1917_01684	-1.2908	Diaminopimelate epimerase
196	BF1917_01690	-1.3927	Guanosine-5'-triphosphate%2C3'-diphosphate pyrophosphatase
197	BF1917_01709	-0.9715	Thioredoxin-1
198	BF1917_01711	-2.0211	5-methyltetrahydropteroyltriglutamate--homocysteine methyltransferase
199	BF1917_01757	-1.3548	hypothetical protein
200	BF1917_01771	-0.8441	ECF RNA polymerase sigma factor SigG
201	BF1917_01800	-0.9265	Quercetin 2%2C3-dioxygenase
202	BF1917_01920	-1.2429	Toluene efflux pump periplasmic linker protein TtgD precursor
203	BF1917_01957	-1.2235	putative oxidoreductase/MSMEI_2347
204	BF1917_01960	-1.3016	Oxygen-insensitive NAD(P)H nitroreductase
205	BF1917_01989	-1.1508	Pyruvate dehydrogenase [ubiquinone]
206	BF1917_02034	-1.2158	bacterioferritin
207	BF1917_02045	-1.0975	Cupin domain protein
208	BF1917_02050	-1.0708	hypothetical protein
209	BF1917_02051	-1.4625	hypothetical protein
210	BF1917_02058	-1.2098	2-oxoglutarate carboxylase small subunit
211	BF1917_02059	-0.9484	Methylmalonyl-CoA carboxyltransferase 1.3S subunit
212	BF1917_02075	-1.4466	hypothetical protein
213	BF1917_02077	-1.0832	Beta-lactamase type II precursor
214	BF1917_02080	-1.5189	hypothetical protein
215	BF1917_02082	-1.3791	hypothetical protein
216	BF1917_02083	-1.3787	Phosphotransferase RcsD
217	BF1917_02086	-1.0143	Membrane-bound lytic murein transglycosylase D precursor
218	BF1917_02095	-1.143	Peptide methionine sulfoxide reductase MsrA/MsrB
219	BF1917_02111	-1.0889	Pyridoxine kinase
220	BF1917_02113	-1.6011	Lactate utilization protein B
221	BF1917_02114	-1.2751	Lactate utilization protein A
222	BF1917_02120	-0.9187	hypothetical protein
223	BF1917_02166	-1.5855	Anaerobic ribonucleoside-triphosphate reductase
224	BF1917_02197	-1.8031	Outer membrane efflux protein
225	BF1917_02219	-1.4523	4-hydroxy-3-methylbut-2-enyl diphosphate reductase
226	BF1917_02244	-1.662	Choloylglycine hydrolase
227	BF1917_02276	-1.4009	Ribonucleoside-diphosphate reductase NrdZ
228	BF1917_02308	-0.9336	Glutathione peroxidase homolog BsaA
229	BF1917_02334	-1.3017	P-protein
230	BF1917_02414	-1.5256	Outer membrane protein 40 precursor

231	BF1917_02469	-2.1202	tetratricopeptide repeat protein
232	BF1917_02470	-1.2335	PBP superfamily domain protein
233	BF1917_02471	-2.365	Gram-negative bacterial tonB protein
234	BF1917_02472	-2.9219	Biopolymer transport protein ExbD/TolR
235	BF1917_02473	-2.8621	Biopolymer transport protein ExbD/TolR
236	BF1917_02474	-2.5669	colicin uptake protein TolQ
237	BF1917_02536	-1.0238	hypothetical protein
238	BF1917_02537	-1.1719	hypothetical protein
239	BF1917_02538	-1.1514	hypothetical protein
240	BF1917_02567	-1.0561	Anaerobic C4-dicarboxylate transporter DcuA
241	BF1917_02568	-1.6947	L-asparaginase 2 precursor
242	BF1917_02569	-1.7391	hypothetical protein
243	BF1917_02659	-1.1812	Rhomboid family protein
244	BF1917_02707	-1.5013	FKBP-type 22 kDa peptidyl-prolyl cis-trans isomerase
245	BF1917_02722	-1.049	hypothetical protein
246	BF1917_02802	-1.4168	Putative NAD(P)H-dependent FMN-containing oxidoreductase YwqN
247	BF1917_02803	-0.8956	NADP-dependent alcohol dehydrogenase C 2
248	BF1917_02905	-1.5008	ISXO2-like transposase domain protein
249	BF1917_02906	-1.3578	hypothetical protein
250	BF1917_02914	-0.8332	hypothetical protein
251	BF1917_02930	-1.5235	fermentation/respiration switch protein
252	BF1917_02976	-1.051	hypothetical protein
253	BF1917_02993	-1.1518	LexA repressor
254	BF1917_03013	-0.819	Iron-binding zinc finger CDGSH type

Note: P-adjusted value ≤ 0.05

Table S2: PCR primers for Phage genes identified by RNA-seq

Gene	Name	Sequence
CDR20291_1208	CDR20291_1208_FW	atatatAGGCTCCAACACCAAAAGAA
	CDR20291_1208_REV	cgcgcgACCATAACCCATAACATCTTGAAGT
CDR20291_1436	CDR20291_1436_FW	atatatACAAAAGTTTCCAGAGATGGATGC
	CDR20291_1436_REV	cgcgcgTGCTGTGTTACCTAATGCGGT
Universal 16S rRNA bacterial primers	27F	AGAGTTTGATCCTGGCTCAG
	1392R	GGTTACCTTGTTACGACTT
